# Can the Wild Perennial, Rhizomatous Rice Species* Oryza longistaminata* be a Candidate for De Novo Domestication?

**DOI:** 10.1186/s12284-023-00630-7

**Published:** 2023-03-16

**Authors:** Shuai Tong, Motoyuki Ashikari, Keisuke Nagai, Ole Pedersen

**Affiliations:** 1 Department of Biology, University of Copenhagen, Universitetsparken 4, 3Rd Floor, 2100 Copenhagen, Denmark; 2 Bioscience and Biotechnology Center of Nagoya University, Furo-Cho, Chikusa, Nagoya, Aichi 464-8602 Japan; 3 School of Agriculture and Environment, The University of Western Australia, 35 Stirling Highway, Crawley, WA 6009 Australia

**Keywords:** Abiotic stress, Drought tolerance, Flood tolerance, Genome editing, Heat tolerance, Perennial, Rhizome, Salinity tolerance, Submergence tolerance

## Abstract

As climate change intensifies, the development of resilient rice that can tolerate abiotic stresses is urgently needed. In nature, many wild plants have evolved a variety of mechanisms to protect themselves from environmental stresses. Wild relatives of rice may have abundant and virtually untapped genetic diversity and are an essential source of germplasm for the improvement of abiotic stress tolerance in cultivated rice. Unfortunately, the barriers of traditional breeding approaches, such as backcrossing and transgenesis, make it challenging and complex to transfer the underlying resilience traits between plants. However, de novo domestication via genome editing is a quick approach to produce rice with high yields from orphans or wild relatives. African wild rice, *Oryza longistaminata*, which is part of the AA-genome *Oryza* species has two types of propagation strategies viz. vegetative propagation via rhizome and seed propagation. It also shows tolerance to multiple types of abiotic stress, and therefore *O. longistaminata* is considered a key candidate of wild rice for heat, drought, and salinity tolerance, and it is also resistant to lodging. Importantly, *O. longistaminata* is perennial and propagates also via rhizomes both of which are traits that are highly valuable for the sustainable production of rice. Therefore, *O. longistaminata* may be a good candidate for de novo domestication through genome editing to obtain rice that is more climate resilient than modern elite cultivars of *O. sativa*.

## Introduction

Future challenges in crop production are unparalleled as the human population will exceed 10 billion by 2050 (FAO 2017). However, while staple crops and livestock demand are predicted to increase by 60% by 2050 (Springmann et al. 2018), increases in production have a history of stagnating or even decreasing over time as land degradation results in huge loss of arable land (Grassini et al. 2013). Moreover, higher yields are also required in order to counteract climate change, which is forecasted to severely restrict plant production due to intensified abiotic stress. Therefore, the development of crops that can tolerate abiotic stresses such as flooding, drought, salinity, heat and cold is needed to grow crop production (López-Marqués et al. 2020), including rice that accounts for 20% of the world’s calorie production (Pandey et al. 2010). Fortunately, species of wild rice exhibit an astonishing diversity in morphology, height, tillering, flowering, growth habit, panicle, leaf, culm, and seed characteristics (Ali et al. 2010). Moreover, these plants have 15 million years of evolutionary history, during which numerous ecological adaptations to abiotic stress have evolved (Vaughan et al. 2003), and hence it seems attractive to search for valuable traits among the wild relatives.

Rice has already been domesticated twice from two different progenitors. The first domestication took place in Asia from populations of wild *O. rufipogon* Griff. leading to a new recognized species of *O. sativa* L. with 2 subspecies “*japonica*” and “*indica*” (Cheng et al. 2003). The current combined genetic and geographic analyses provide evidence for multiple domestications of *O. sativa*. Subspecies *japonica* and *indica* appear to have arisen from separate gene pools with *japonica* from populations of *O. rufipogon* in southern China and *indica* from *O. rufipogon* populations in India or Indochina (Londo et al. 2006). In Africa, *O. barthii* A. Chev. has been domesticated leading to the species of *O. glaberrima* Steud., i.e., African rice (Sarla and Swamy 2005; Sweeney and McCouch 2007; Wang et al. 2014). However, the process of domestication of *O. glaberrima* has also not been fully established and it is therefore not clear if multiple populations of *O. barthii* have been domesticated in different regions of Africa, or if the domestication process has only taken place once (Wambugu et al. 2021). On both continents, early hunter-gatherers and ancient farmers have selected for loss of function of undesirable agronomic traits such as seed shattering and lodging controlled by, e.g., *sh4* (shattering) (Li et al. 2006) and *prog1* (lodging) (Wang and Li 2008). Additional factors such as the widespread adoption of high-yielding elite cultivars in combination with a change in farming systems, industrialization and consumers’ preferences for certain traits have further led to erosion of the rice gene pool so that cultivated rice now show significantly lower genetic diversity compared to its wild ancestors (Sun et al. 2001). The lower genetic diversity potentially renders cultivated rice vulnerable to climate change if key genes coding for tolerance to abiotic stress have been lost from its gene pool. Fortunately, numerous species of wild plants have evolved several mechanisms to protect themselves from environmental stresses, but it is typically challenging and complex to transfer the underlying resilience traits to our modern, high-yielding crops.

In addition to the two cultivated species of rice, the *Oryza* genus contains 21 species of wild rice (Vaughan et al. 2003). The habitat preferences of wild rice spans from wetlands to drylands and from fresh to saline soils and consequently, the rich genetic pool within *Oryza* holds traits conferring tolerance to many types of abiotic stress. A study using a Geographic Information System (GIS) approach where georeferenced occurrences of wild rice species were overlaid by environmental maps identified several candidate species that should be further explored in the search for tolerance to heat (five species), cold (one species), drought (five species) or flooding (four species) (Atwell et al. 2014). The rich genetic pool of wild rice has already been successfully employed to improve abiotic stress tolerance or disease tolerance of cultivated rice with well-known examples from, e.g., *O. rufipogon, O. nivara* S.D.Sharma & Shastry, *O. officinalis* Wall. ex Watt and *O. perennis* Moench that have been used to introgress bacterial blight resistance, blast resistance, brown plant hopper resistance and cytoplasmic male sterility resistance, respectively (Brar and Khush 2018). Regrettably, the genetic pool of species of wild rice is rapidly shrinking due to habitat loss leading to dramatic declines, or even eradication (Akimoto et al. 1999), in populations size as exemplified by populations of *O. rufipogon* in China (Lu and Sharma 2003; Song et al. 2005). Hence, tapping into this rich source of genetic variation is a race against time as the demand for resilient rice is increasing while the supply from the natural germplasm is declining.

The genus of *Oryza* consists of 11 genomes with cultivated rice (*O. sativa* and *O. glaberrima*) belonging to the AA genome (Vaughan 1994). The six wild species within the AA genome are easily crossed with both species of cultivated rice, and 66% of the successful introgression of candidate traits from wild relatives is therefore based on species from the AA genome (Brar and Khush 2018). Here, backcross breeding, where elite cultivars are crossed with wild relatives possessing the desired trait followed by multiple crossings of the hybrid with its parent, has been the main approach used to develop new stress-tolerant varieties (Sharma et al. 2021; Vogel 2009). However, backcross breeding is time-consuming, it works poorly with quantitative or recessive traits, and it is often further complicated by sexual barriers (Kushwah et al. 2020). Alternatively, transgenesis, which is independent of the crossing ability of the parent plants, has been predicted to be used for the majority of key crops in the future (Tester and Langridge 2010). However, transgenic plants are considered GMOs (genetically modified organisms) and are banned from many markets around the world due to consumer concerns (Buchholzer and Frommer 2023), and consequently an alternative fast-forward breeding approach to accelerate rice domestication and create climate-resilient rice is needed (Marsh et al. 2021). One such approach could be de novo domestication of wild rice relatives.

De novo domestication of rice is the process of introduction of domestication traits via mutagenesis into wild rice species. Hence, rather than incorporating the desired traits from wild species into modern elite cultivars, de novo domestication is conducted to mimic the natural process of evolution (Fernie and Yan 2019). In de novo domestication, undesirable traits in the wild rice species are deleted by genome editing, while preserving the beneficial genes controlling stress resilience and agronomically important traits that had disappeared in the process of domestication of *O. rufipogon* or *O. barthii* (Gasparini et al. 2021). The domestication of wild rice into attractive new rice may now be accomplished in a few generations due to the rapidly growing genome editing toolbox, when in the past traditional domestication has typically taken hundreds of years (DeHaan et al. 2020; Eshed and Lippman 2019). In fact, rice is currently a showcase for this promising approach with a recent study demonstrating that six agronomically important traits of *O. alta* Swallen (tetraploid wild rice belonging to the CCDD genome) can be rapidly modified using genome editing (Yu et al. 2021).

**Fig. 1:**
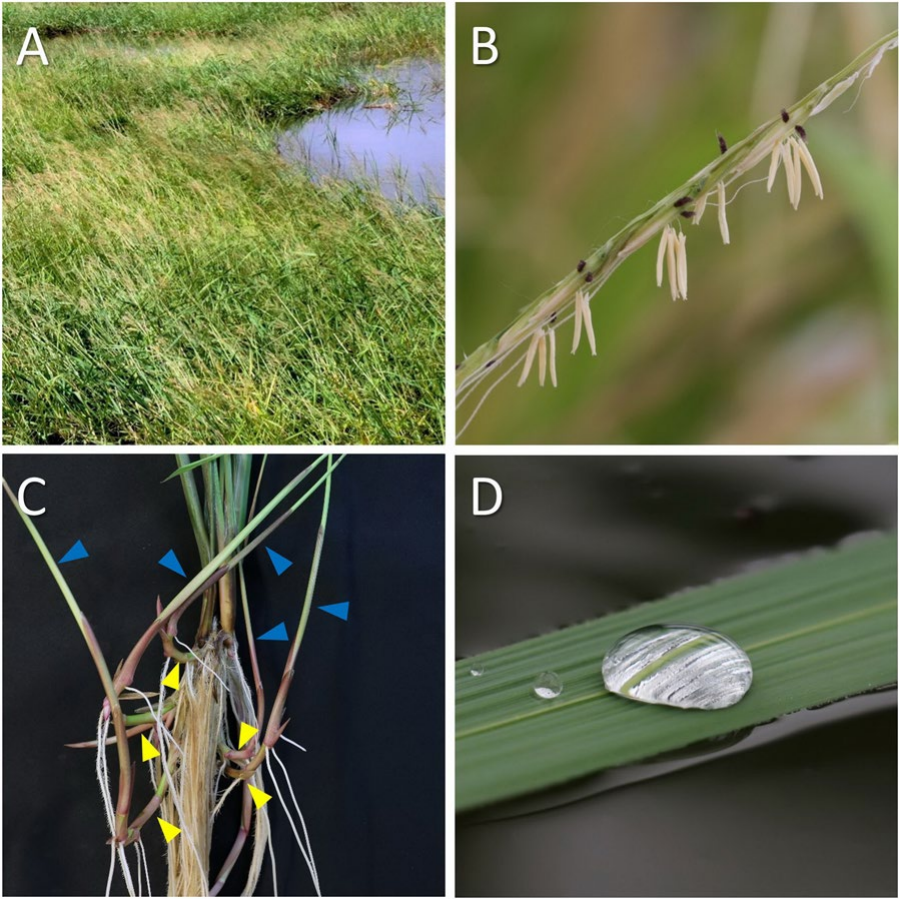
Habitat photo (**A**) of *Oryza longistaminata* from Madagascar where it forms a dense stand in a natural wetland. The flower (**B**) is characterized by its very long stamens, and it is the only rhizome-bearing species in the AA genome; horizontal rhizomes are indicated by yellow arrowheads and blue arrowheads indicate vertical ramets (**C**). The leaves are superhydrophobic (**D**) and retain a thin gas film during submergence facilitating gas exchange (CO_2_ and O_2_) with the floodwater (Colmer and Pedersen 2008). Photos by Jean-Augustin Randriamampianina (**A**) or the authors (**B**-**D**)

In this paper, we discuss the prospects of de novo domestication of *O. longistaminata* A.Chev.& Roehr., a species of wild rice belonging to the AA genome and with several useful traits vested in its natural genome (Fig. 1). *O. longistaminata* has been identified as a candidate species for both heat and drought tolerance and its potential temperature plasticity has also been highlighted (Atwell et al. 2014). Interestingly, *O. longistaminata* served as donor for the *Xa21* gene conferring tolerance to bacterial blight, which was one of the first genes being introgressed from wild rice species into IR24 (*O. sativa ssp. indica*), an IRRI genotype (Khush et al. 1990). Moreover, *O. longistaminata* is perennial and forms rhizomes both of which are valuable traits (Getachew et al. 2020; Hu et al. 2011). We propose working more extensively with this exciting species native to Africa in order to evaluate if it holds a strong potential for de novo domestication with resilience to abiotic stress.

## *O. longistaminata –* A Genetic Resource with Some Undesirable Traits

*O. longistaminata* is native to Africa and the only species in the AA-genome of *Oryza*, which is both perennial and propagates via rhizomes (Getachew et al. 2020; Hu et al. 2011; Vaughan 1994; Vaughan et al. 2003). These are both highly desirable agronomic traits in the future of rice production, where the consumers will demand more sustainable rice production, which can be facilitated by growing perennial rice with rhizomes. However, there are also some undesirable traits in *O. longistaminata*, which would need elimination before this species represents an attractive alternative to the modern elite varieties of *O. sativa* or *O. glaberrima*.

Among these undesirable traits is the inherently low productivity of *O. longistaminata* caused by self-incompatibility. Self-incompatibility reduces inbreeding (Takayama and Isogai 2005) and is rare in rice, but in *O. longistaminata* it is particularly pronounced (Zhang et al. 2015). This species already forms large clones due to vegetative growth via rhizomes, and in this particular case, self-pollination would locally lead to little genetic diversity. From an evolutionary point of view, it therefore makes sense if self-incompatibility is favoured for a plant that is capable of colonizing via clonal growth (Vallejo-Marín and O’Brien 2007). Nevertheless, the self-incompatibility needs to be eliminated in order to secure grain filling and thereby form an attractive alternative to current rice cultivars. The issue of self-incompatibility has already been studied in *O. longistaminata* and it seems that one gene (*Olong01m10012815*) is highly upregulated in the pistils of the self-compatible hybrid between *O. longistaminata* and *O. sativa* (Zhang et al. 2015). Interestingly, this gene is located in the same region where the gene for self-incompatibility has been identified in perennial ryegrass (Yang et al. 2009). However, studies targeting the exact genetic network responsible for self-incompatibility in *O. longistaminata* are needed in order to tackle its low grain productivity.

Another highly undesirable agronomical trait needing elimination in *O. longistaminata* is its inherent tendency for seed shattering. The many species of wild rice disperse their seeds freely at maturity to maximize sexual propagation (Maity et al. 2021), and the early farmers selected strongly against this trait in order to enable harvesting the grain at maturity (Ishikawa et al. 2022). Some studies indicate that the non-shattering trait was selected for very early in the history of domestication and possibly even before the *indica-japonica* differentiation (Lin et al. 2007). The genetic network involved in seed shattering in rice is fairly well described (Konishi et al. 2006; Li et al. 2006). The first gene reported coding for shattering is the *sh4* (Li et al. 2006), and all of the domesticated cultivars of *O. sativa* (92 *indica* and 108 *japonica*) included in a subsequent study possessed a mutation in *SH4* caused by only a single amino acid substitution in contrast to all of the tested wild rice accessions (24 in total) with none of them possessing the mutation (Lin et al. 2007). A very recent study, however, has shown that the interruption of the abscission layer formation requires mutation of both *sh4* and *qSH3* demonstrating that the selection process against shattering in rice was not as simple as previously suggested (Inoue et al. 2015; Ishikawa et al. 2022). Nevertheless, the well-described genetic network involved in seed shattering increases the chance of successful genome editing resulting in plants where the mature seeds are retained in the panicle enabling harvesting.

Consequently, if the undesirable traits of *O. longistaminata* – including the major ones outlined above – can be successfully tackled using modern genome editing, the higher genetic diversity of *O. longistaminata* suggests that it is a good candidate species for traits involved in abiotic stress tolerance and possibly also tolerance to pest (Getachew et al. 2020). In fact, resistance to bacterial blight disease conferred by *Xa21* was first discovered in *O. longistaminata* (Khush et al. 1989) and then subsequently introgressed into modern cultivars, and therefore this species has already demonstrated its usefulness within disease tolerance. Below, we discuss the potential benefits of the two major habits of *O. longistaminata*, i.e., its perennial growth and the formation of rhizomes, and we also identify possible tolerances to abiotic stress.

## Perennial Rice

Numerous environmental issues, including land degradation, water pollution, and greenhouse gas emissions, are linked to modern agriculture derived from the predominant use of annual crops (Crews et al. 2018). The annual clearing of vegetation causes soil erosion and subsequent leakage of valuable nutrients to both groundwater and surface waters (Cox et al. 2010). In contrast to annual crops, perennials retain a substantial proportion of the nutrients that were taken up during the growth season (Thorup-Kristensen et al. 2009). The nutrients are retained in roots and belowground stems preventing loss to the environment and also reducing fertilizer requirements in the following season (Kawai et al. 2022). Moreover, perennial crops address carbon depletion of agricultural soils by increasing soil carbon storage, thereby restoring soil function, and buffering the ongoing increase in atmospheric CO_2_ (Poeplau et al. 2015; Robertson et al. 2000). Perennial crops therefore have the potential to contribute to the protection of biodiversity, through reduced agricultural inputs resulting in improved water quality, higher carbon sequestration, and tighter nutrient cycles, without negative impact on crop productivity. Consequently, the creation of perennial cereals is now often proposed as a key strategy for the development of sustainable agriculture (Glover et al. 2007).

The perennial rice breeding program of the International Rice Research Institute (IRRI), which operated from 1995 to 2001, was promising for upland rice. The yield potential of many of the hybrids matched or exceeded the yields of current cultivars (Sacks et al. 2003). More recently, a perennial rice cultivar known as PR23, which was generated by embryo rescuing of crossings of *O. sativa* and *O. longistaminata*, was released for testing under paddy conditions in southern China and Laos (Samson et al. 2018; Zhang et al. 2019). In comparison with the main conventional rice cultivars, PR23 has shown very promising results in the field trials conducted in nine ecological regions of Southern China from 2011 to 2017. PR23 with its perennial habit obtained high yields across sites, among years, and cycles of regrowth, and it was less labour-intensive, and had greater economic returns (Huang et al. 2018). Grain quality was equal to RD23 (one of the annual control cultivars), and milling quality was exceptional so farmers and millers were impressed with PR23 (Huang et al. 2018). More recently, the performance of PR23 has been thoroughly evaluated showing that the cultivar performed equally well to annual rice with sustained yields of 6.8 ton ha^−1^ y^−1^ over a four-year period, and in 2021, PR23 was grown on 15,333 ha by 44,752 smallholder farmers in southern China (Zhang et al. 2021). Moreover, the soil carbon content increased by almost 1 ton ha^−1^ y^−1^, soil nitrogen by 100 kg ha^−1^ y^−1^, and soil pH increased by up to 0.4 units (Zhang et al. 2021) showing the huge environmental benefits associated with perennial rice.

## Rhizome-Bearing Rice

A key trait conferring tolerance to periods of unfavourable environmental conditions is the rhizome. Numerous perennial plant species have rhizomes (Yang et al. 2015), and plants possessing this trait are referred to as rhizomatous, and these can be found in a variety of habitats (Guo et al. 2021). Rhizomes are belowground swollen stems used as storage organs, and they consist of numerous phytomers each containing a piece of internode and a node with an auxiliary bud at the base of the scale leaf; these meristems can develop into new stems some of which form a new rhizome or bend upwards to vertical shoots (ramets) (Bessho-Uehara et al. 2018; Yoshida et al. 2016). At the apical part of the rhizome, the scale leaves help protecting the rhizome as it pushes through the soil. Moreover, adventitious roots form at each node to help supporting nutrient and water acquisition. The clonal integration enables inter-ramet transport of not only nutrients but also photosynthates so that horizontal gradients in resources can be compensated for within the clone (Shibasaki et al. 2021). Therefore, the clonal growth habit can be considered a strategy for long-term survival and vegetative spreading (Hacker 1999). Moreover, due to the storage of reduced carbon and the numerous buds, the rhizome can support rapid clonal re-growth after die-back of the aboveground shoots as caused by a period of abiotic stress. For example, the extensive rhizome network in some *Cynodon dactylon* (bermudagrass) genotypes confers high drought resistance (Zhou et al. 2014), and it has been shown that clonal plants on inland dunes tolerate animal grazing significantly better than non-clonal plants (Liu et al. 2007).

Within the genus of *Oryza*, six species are reported to possess rhizomes viz*. O. australiensis* Domin (AA genome), *O. eichingeri* Peter (CC genome and also known as *O. rhizomatis* D. A. Vaughan), *O. longistaminata* (Mondal and Henry 2018; Vaughan 1994), *O. officinalis* (CC genome) (Vaughan 1994), *O. meyeriana* Baill (GG genome), and *O. coarctata* Roxb (KKLL genome, now moved to the genus of *Porteresia*) (Mondal and Henry 2018). Domesticated rice, sorghum and maize are all grain producing annuals, but oddly enough none of them have the rhizomatous feature. Instead, each of them has a closely related perennial and rhizomatous relative called *O. longistaminata, S. propinquum* or *Zea diploperennis*. The majority of rhizome-forming quantitative trait loci (QTLs) in sorghum and rice show a strong correlation, indicating that some of the same genes may control the rhizomatous trait in these distantly related grass species within the family of Poaceae (Li et al. 2022). This finding supports the hypothesis that cultivated annual sorghum and rice may have arisen from their perennial, rhizomatous ancestors through mutations in related genes (Hu et al. 2003; Kong et al. 2015).

Rhizomes are useful for agriculture, and they are also beneficial for the environment. As discussed above, production of annual crops results in huge losses of valuable nutrients from the belowground organs when the shoot is harvested and the belowground tissues subsequently decay. Moreover, since a new annual crop is started from seeds, herbicide spraying is also required to reduce the fierce competition from weeds when the seedlings are young and competitively inferior (Thorup-Kristensen et al. 2020). In contrast, the rhizome can support deep root systems from stored energy and thereby prevent these from dying, and the deep roots allow new vertical shoots to immediately tap into water and nutrient resources deep in the soil, which is in stark contrast to the shallow roots of young seedlings (Kell 2011). In many grain-producing systems, the deep rooting would save water and fertilizers reducing eutrophication of surface waters, leaching of nitrogen into the groundwater, and reduced water abstraction would ensure environmental flow in steams and an ecosystem-friendly water table in neighbouring lakes and ponds (Arthington et al. 2006). *O. longistaminata* belongs to the AA genome and can be crossed with *O. sativa* producing offspring with fertile seeds, and therefore it is perhaps not surprising that it has been nominated as ideal research material to unravel the mechanisms controlling rhizome development (He et al. 2014).

## Abiotic Stress Tolerance in *O. longistaminata*

**Fig. 2:**
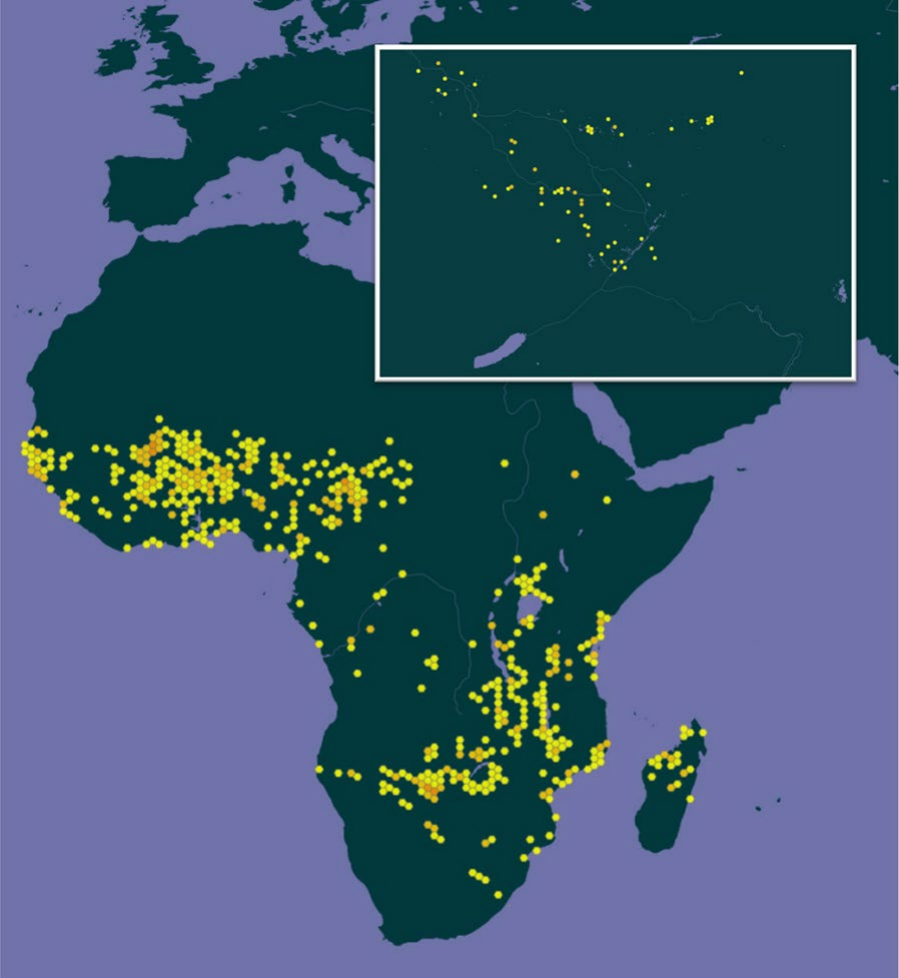
2,634 geo-referenced occurrences of *Oryza longistaminata*. Lightly coloured hexagons indicate few observations whereas darker hexagons indicate numerous observations. The insert shows the Okavango Delta where *O. longistaminata* is found in high densities. Data were extracted from www.Gbif.org in December 2022

*O. longistaminata* is growing in a range of contrasting habitats, and it grows to a height of more than 2 m and propagates year-round through its extensive rhizome network (Bessho-Uehara et al. 2018). It is sometimes referred to as red rice or long-stamen rice, where the latter name derives from its unusually long stamens (Fig. 1B). *O. longistaminata* is endemic to Africa and occurs south of Sahara with most observations from West Africa, the southern part of East Africa and Madagascar, but this species is also widely distributed around the Okavango Delta (Fig. 2). With more than 2,500 georeferenced observations, *O. longistaminata* is an excellent candidate for GIS-based habitat classification using the approach of Atwell et al. (2014). Using this approach, high-resolution environmental maps of, e.g., soil moisture, soil pH, soil salinity or sodicity can be used to identify target populations with promising adaptation to abiotic stress.

GIS-based habitat classification has previously been used also for *O. longistaminata*, and this species was singled out as a key candidate for drought and heat stress tolerance (Atwell et al. 2014). Based on the distribution in temperature and moisture extremes, *O. longistaminata* was considered a key candidate for heat tolerance and is also likely to be a candidate for drought tolerance (Atwell et al. 2014). *O. longistaminata* has thick leaves and high mesophyll conductance to CO_2_ diffusion, suggesting it may be drought tolerant given that these traits are linked to higher water use efficiency (Giuliani et al. 2013).

Interestingly, Fig. 2 clearly indicates that *O. longistaminata* occurs in coastal regions indicating that some populations likely harbours genetic resources coding for salinity tolerance. Therefore, we propose repeating the study of Atwell et al. (2014) with inclusion of relevant environmental maps of even higher resolution in an attempt to identify promising populations of *O. longistaminata*, which can be sampled and analysed further for abiotic stress tolerance under controlled laboratory conditions.

Indeed, a very recent study identified 18 QTLs for salinity tolerance in *O. longistaminata* (Yuan et al. 2022). On chromosome 2, a QTL for salt injury score, the water content of seedlings treated with salt, and relative water content of seedlings were repeatedly found and co-localized. Based on sequence and expression analysis, a cytochrome P450 86B1 (*MH02t0466900*) was proposed as a potential candidate gene for salt tolerance, and these results have established the groundwork for future molecular breeding efforts to further enhance rice salt tolerance.

A significant issue in cereal crops is lodging, which lowers grain yield and grain quality (Shah et al. 2017). To overcome this obstacle, numerous efforts have been made to develop lodging-resistant cultivars of rice, maize, and other crops (Yadav et al. 2017). Generous use of fertilizer produces tall, lodging-prone rice plants with decreased yield, and it is therefore crucial to identify QTLs or genes coding for lodging resistance in order to improve the germplasm of modern elite cultivars. The conspicuous strong stems and good biomass productivity in *O. longistaminata* make it a potential candidate gene pool to improve lodging resistance. Stem diameter, stem length, and breaking strength were all significantly enhanced by a QTL called *qLR1*, which was located in an area of 80 kb on chromosome 1. Moreover, the breaking strength was greatly increased by another QTL, *qLR8*, which was located on chromosome 8 and defined in a region of about 120 kb (Long et al. 2019). These findings demonstrate that *O. longistaminata* can be used to create rice varieties that are resistant to lodging and if used for de novo domestication, these crucial genes are already present in the germplasm.

Submergences stress is another type of abiotic stress, which has been predicted to increase in rice-growing areas with the ongoing climate changes. Rice can respond to submergence in two contrasting ways by *i*) stem elongation to keep track with the rising floodwaters so that the shoot can act as a snorkel (Bailey-Serres and Voesenek 2008; Colmer and Voesenek 2009) or *ii*) repressing elongation in order to save carbohydrates and wait for the floodwater to recede (Colmer and Voesenek 2009). The first type of response is known from deepwater rice (Hattori et al. 2009; Kende et al. 1998; Kuroha et al. 2018; Nagai et al. 2020), whereas the latter was first discovered in an Indian landrace (FR13A) and later the relevant genes were introgressed into elite cultivars, which have been adopted by farmers in SE Asia (Xu and Mackill 1996; Xu et al. 2006). To our knowledge, it is not yet known if *O. longistaminata* utilizes the first or the second strategy when exposed to submergence stress; it may even employ a third and yet unknown response to submergence.

During submergence, the exchange of O_2_ and CO_2_ with the floodwater is greatly restricted due to the 10^4^-fold slower diffusion of gasses in water compared to in air. However, the superhydrophobic leaves of *O. sativa* retain a thin leaf gas film upon submergence enabling the stomata to still operate. The gas film prevents flooding of the sub-stomatal cavity and it presents a large surface area for gas exchange with the floodwater (Pedersen et al. 2009; Verboven et al. 2014). Thereby, underwater photosynthesis and underwater respiration can be sustained during complete submergence (Colmer and Pedersen 2008). There are no studies on underwater photosynthesis or respiration in *O. longistaminata*, but Fig. 1D clearly suggests that the leaves of *O. longistaminata* are superhydrophobic (indicated by the silvery sheen below the water droplets) so that these would retain a leaf gas film during submergence. However in *O. sativa*, the hydrophobicity is lost during time of submergence (Winkel et al. 2014) and we therefore propose to investigate if *O. longistaminata* retains its superhydrophobicity during submergence as this would make it an excellent candidate to further improve submergence tolerance of cultivated rice.

## Genome Editing of *O. longistaminata*

Isolation and breeding application of specific genes involved in stable production, such as stress tolerance in rice, have been actively conducted for decades. However, stress tolerance is generally controlled by a large number of quantitative trait loci, and the usual breeding strategy is to gradually increase tolerance traits by pyramiding individual tolerance genes (Singh et al. 2021). In other words, the strategy is to gradually enhance stress tolerance by building on the very limited genetic diversity of existing varieties and conventional lines. Using this conventional approach, the available diversity is limited, and it is difficult to confer significant stress tolerance such as salt-tolerance (Singh et al. 2021). In addition, even with methods such as marker selection breeding, breeding crops with sufficient tolerance takes time, and there is no guarantee that this can be achieved. It is therefore uncertain if conventional breeding methods will be sufficient to cope with the rapid environmental changes that humanity is facing in the future.

Rice is naturally equipped with mechanisms whereby DNA is promptly repaired following damages caused by, e.g., ultraviolet light. Occasionally, accidental deletion, insertion, or substitution of nucleotides may occur, and spontaneous mutations thereby appear, leading to the inability of a particular gene to function. The genome editing technology can introduce mutations into DNA by cutting arbitrary points on the genome using artificial nucleases that serve as scissors, such as CRISPR/Cas9 (Gaj et al. 2013). This makes it possible to introduce mutations in coding regions to affect the function of specific proteins (fx. enzymes, transporters, or receptors). Another major advantage is the ability to select individuals that do not carry the CRISPR/Cas9 expression cassette (null segregants, non-native introduced genes) by Mendelian segregation in later generations, and to genetically fix the introduced mutation (Xu et al. 2015). There are currently three major categories of genome editing technologies. *i*) Site-directed nuclease (SDN)1, where the mutation is introduced during spontaneous repair after cleavage of a host target sequence by an artificial nuclease. *ii*) SDN2, where the mutation of a few nucleotides in a specific region of the genome is introduced by homologous recombination with an extracellularly processed template DNA sequence after cleavage by an artificial nuclease. *iii*) SDN3, where a larger number of nucleotides are introduced into the genome by the same method as SDN2, e.g., the entire length of the gene. In many countries, it is currently discussed whether plants produced by each of these technologies should be considered GMOs (Buchholzer and Frommer 2023).

Genome editing has already been used to modify agronomically important traits of a wild *Oryza* species. Important traits such as lodging resistance, heading date, and grain size were successfully modified in *O. alta* demonstrating a potential path forward for creating stress-resistant rice by combining genomics knowledge of cultivated crops, desirable traits found in wild rice species, and rapid genetic change via genome editing (Yu et al. 2021).

**Fig. 3:**
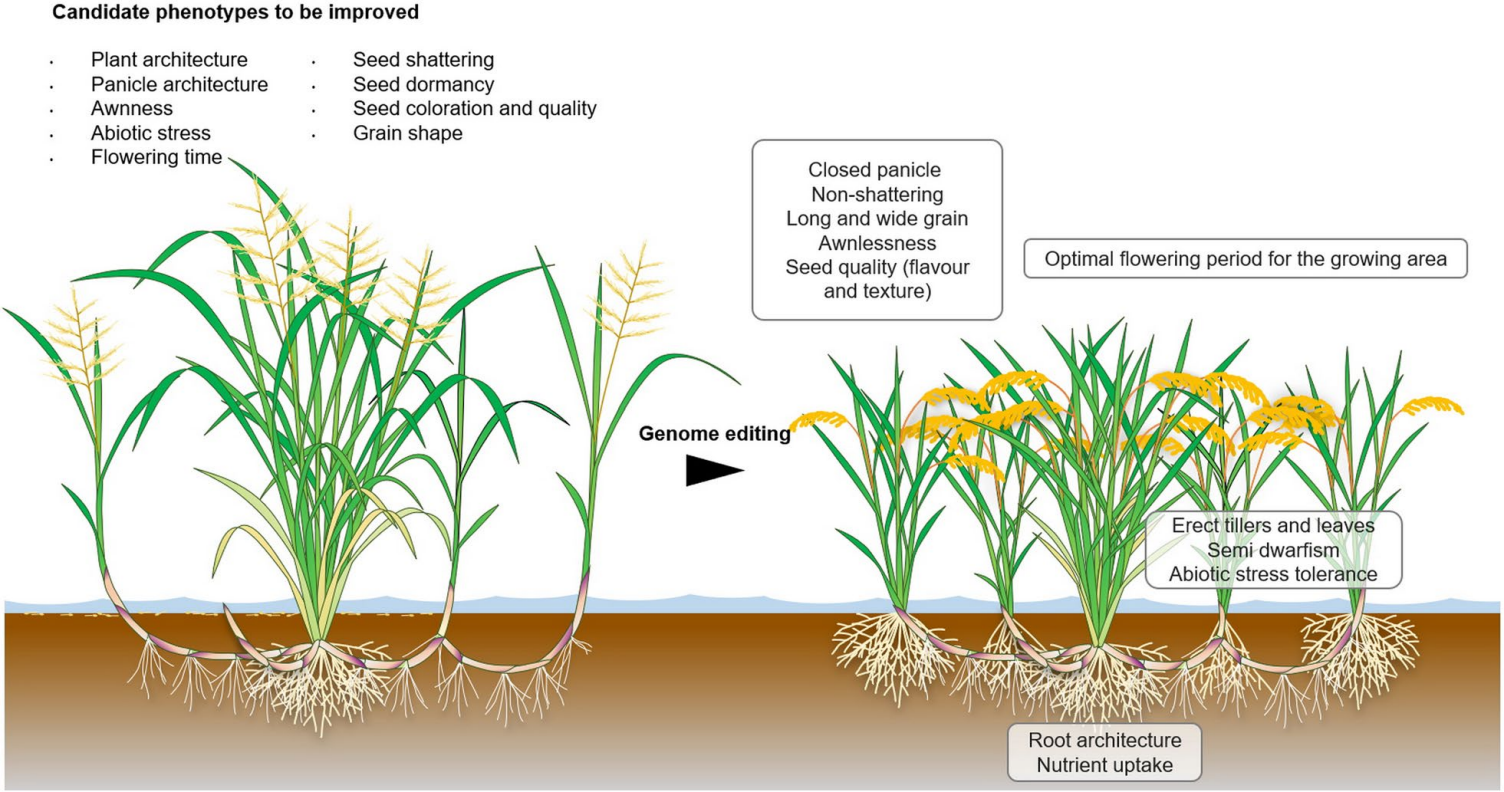
The pathway from wild *Oryza longistaminata* to novel de novo domesticated *O. “toleransa”* using genome editing. Target genes are listed in Tables 1, 2 and 3 in order of priority from essential silencing of highly undesirable genes over genes used to target specific environmental conditions to enhancing expression of genes resulting in attractive genotypes

In the case of *O. longistaminata*, numerous traits need to be edited in order to produce agronomically attractive genotypes (Fig. 3). Several major genes have been identified, many of which confer cultivated traits through functional deletion or loss of function. Therefore, we have identified candidate genes known from *O. sativa* and *O. glaberrima* that are likely to confer cultivation traits also in *O. longistaminata* by genome editing. The first set of genes requires editing in order to produce agronomically acceptable phenotypes. Rice is a short-day plant that shifts to reproductive growth under short-day conditions. In the de novo domestication of *O. longistaminata*, we have identified candidate genes for the control of tiller number, loss of shattering, loss of awn, seed size, and abiotic stress tolerance (Table 1).

**Table 1:** Key candidate genes to be knocked out in *Oryza longistaminata* in order to construct phenotypes with high agronomic value

Trait	Gene name	CGSNL^1^ gene name or gene name synonym(s)	RAP^2^ ID	MSU^3^ ID	Description	Similarity and gaps to *O. sativa* [or haplotype]	References
Plant architecture	*SD1*	*SEMI DWARF 1*	*Os01g0883800*	*LOC_Os01g66100*	GA 20-oxidase2 Highly active GR type (Kuroha et al. 2018)	98.23% (388/395), 1.52% (6/395)	Asano et al. (2007), Sasaki et al. (2002)
Plant architecture	*PROG1*	*PROSTRATE GROWTH 1*	*Os07g0153600*	*LOC_Os07g05900*	Zinc-finger nuclear transcription factor. Deletion of tandem repeat genes (7/8) promoted erect growth and high yield of Asian cultivated rice	[*O. longistaminata* possesses 10 zinc-finger genes]	Jin et al. (2008), Tan et al. (2008), Wu et al. (2018)
Plant architecture	*TAC1* (*SPK*)	*Tiller angle control 1* (*SPREADING STUB of “Kasalath”*)	*Os09g0529300*	*LOC_Os09g35980*	Unknown functionSplicing site of 3'UTR of *O. longistaminata* is indica *TAC1* type (high expression type)	98.84% (256/259), 0%	Huang et al. (2016), Yu et al. (2007)
Plant architecture	*D10* (*OsCCD8*)	*DWARF10* (*carotenoid cleavage dioxygenase 8*)	*Os01g0746400*	*LOC_Os01g54270*	Carotenoid cleavage dioxygenase 8, strigoractone (SL) biosynthesis	98.07% (559/570), 0.53% (3/570)	Minakuchi et al. (2010)
Plant architecture	*OsFC1* (*TB1*)	*FINE CULM 1* (*teosinte-branching 1*)	*Os03g0706500*	*LOC_Os03g49880*	TCP family transcription factor. FC1 function is required for SL to exert its effect of inhibiting bud outgrowth	97.55% (318/326), 1.53% (5/326)	Minakuchi et al. (2010)
Plant architecture and yield	*OsPDCD5*	*Programmed cell death 5*	*Os05g0547850*	*LOC_Os05g47446*	Homolog of the mammalian programmed cell death protein 5	99.22% (128/129), 0%	Dong et al. (2021)
Shattering	*qSH1*	*Shattering (QTL)-1*	*Os01g0848400*	*LOC_Os01g62920*	BEL1-type homeobox family	98.37% (605/615), 0.98% (6/615)	Konishi et al. (2006), Sheng et al. (2020)
Shattering	*Sh4*	*SHATTERING 4*	*Os04g0670900*	*LOC_Os04g57530*	Trihelix transcription factor, Myb/SANT-LIKE 23	96.71% (382/395), 1.77% (7/395)	Li et al. (2006)
Shattering	*Sh5*	*SHATTERING 5*	*Os05g0455200*	*LOC_Os05g38120*	BELL1-type homeodomain transcription factor	97.94% (570/582), 1.20% (7/582)	Yoon et al. (2014)
Shattering	*SHAT1*	*SHATTERING ABORTION 1*	*Os04g0649100*	*LOC_Os04g55560*	APETALA2 transcription factor	97.01% (455/469), 1.71% (8/469)	Zhou et al. (2012)
Seed dormancy	*Sdr4*	*SEED DORMANCY 4*	*Os07g0585700*	*LOC_Os07g39700*	Zinc finger protein	96.73 (325/336), 1.79% (6/336)	Sugimoto et al. (2010)
Awn	*LABA1* (*LOGL6/An-2*)	*LONG AND BARBED AWN 1* (*LOG-like 6/Awn-2*)	*Os04g0518800*	*LOC_Os04g43840*	Cytokinin synthesis enzyme	100% (251/251), 0% (0/251)	Hua et al. (2015)
Awn	*RAE2* (*OsEPFL1*)	*REGULATOR OF AWN ELONGATION 2* (*EPIDERMAL PATTERNING FACTOR-LIKE 1*)	*Os08g0485500*	*LOC_Os08g37890*	Epidermal patterning factor-like 1 (EPFL1) protein, small secretary signal peptide *O. longistaminata* possesses *O. glaberrima* type (6C)	(vs. *O. glaberrima*) 90.77% (118/130), 6.92% (9/130)	Bessho-Uehara et al. (2018)
Seed colouration	*Phr1* (*BHC*)	*Phenol reaction 1* (*BLACK HULL C*)	*Os04g0624500*	*LOC_Os04g53300*	Polyphenol oxidase	95.59% (563/589), 3.23% (19/589)	Yu et al. (2008)
Seed coat colouration	*Rc*	*-*	*Os07g0211500*	*LOC_Os07g11020*	Basic helix-loop-helix (bHLH) protein, proanthocyanidin synthesis	(vs *O. rufipogon*) 96.76% (656/678), 1.92% (12/678)	Konishi et al. (2008), Sweeney et al. (2006)
Seed coat colouration	*Rd*	*RED PERICARP AND SEED COAT*	*Os01g0633500*	*LOC_Os01g44260*	Dihydroflavonol-4-reductase (DFR), proanthocyanidin synthesis	98.25% (280/285), 1.95% (3/285)	Konishi et al. (2008)
Seed quality	*Chalk5*	*-*	*Os05g0156900*	*LOC_Os05g06480*	Vacuolar H^+^-translocating pyrophosphatase	98.19% (760/774), 1.29% (10/774)	Li et al. (2014)
Grain shape	*GW2*	*GRAIN WEIGHT 2*	*Os02g0244100*	*LOC_Os02g14720*	RING-type E3 ubiquitin ligase	99.76% (423/424), 0% (0/424)	Song et al. (2007)
Grain shape	*GL3.1*	*GRAIN LENGTH 3.1*	*Os03g0646900*	*LOC_Os03g44500*	Protein phosphatase with Kelch-like repeat domain	100% (1004/1004), 0% (0/1004)	Zhang et al. (2012)
Grain shape	*TGW3*	*THOUSAND GRAIN WEIGHT 3*	*Os03g0841800*	*LOC_Os03g62500*	GLYCOGEN SYNTHASE KINASE 3/SHAGGY-like family	99.76% (423/424), 0% (0/424)	Ying et al. (2018)
Grain shape	*TGW6*	*TOTAL GRAIN WEIGHT6*	*Os06g0623700*	*LOC_Os06g41850*	Indole-3-acetic acid (IAA)-glucose hydrolase activity	98/86% (346/350), 0% (0/350)	Ishimaru et al. (2013)
Grain shape	*LK3* (*GS3/qGL3a*)	*LONG KERNEL 3 (GRAIN SIZE 3)*	*Os03g0407400*	*-*	Plant-specific organ size regulation (OSR) domain, transmembrane region	92.50% (222/240), 5% (12/240)	Mao et al. (2010)
Grain shape	*APG* (*PIL16*)	*ANTAGONIST OF PGL1* (*PHYTOCHROME INTERACTING FACTOR-LIKE 16*)	*Os05g0139100*	*LOC_Os05g04740*	Typical DNA-binding bHLH protein	88.27% (444/503), 3.38% (17/503)	Heang and Sassa (2012a, b)
Grain shape and panicle architecture	*SG1*	*SHORT GRAIN 1*	*Os09g0459200*	*LOC_Os09g28520*	Unknown product, brassinosteroid signalling	98.10% (155/158), 0.63% (1/158)	Nakagawa et al. (2012)
Panicle architecture	*LG1*	*LIGULELESS 1*	*Os04g0656500*	*LOC_Os04g56170*	SBP (SQUAMOSA promoter Binding Protein) DNA binding protein 8	98.90% (411/416), 0% (0/416)	Ishii et al. (2013), Zhu et al. (2013)
Panicle architecture	*Gn1a*	*GRAIN NUMBER 1A*	*Os01g0197700*	*LOC_Os01g10110*	Cytokinin oxidase/dehydrogenase 2	97.89% (557/569), 1.23% (7/569)	Ashikari et al. (2005)
Panicle architecture	*EP2* (*DEP2*)	*ERECT PANICLE 2 (DENSE AND ERECT PANICLE 2)*	*Os07g0616000*	*LOC_Os07g42410*	Transcriptional regulator	99.05% (1354/1367), 0.15% (2/1367)	Zhu et al. (2010)
Cadmium ion tolerance	*OsCd1*	*-*	*Os03g0114800*	*LOC_Os03g02380*	Major facilitator superfamily protein, Cd uptake	100% (457/457), 0% (0/457)	Yan et al. (2019)
Cadmium ion tolerance	*CAL1*	*CADMIUM ACCUMULATION IN LEAF 1*	*Os02g0629800*	*LOC_Os02g41904*	Defensin-like protein, positive regulation of Cd accumulation in rice leaves	100% (80/80), 0% (0/80)	Luo et al. (2018)
Flooding tolerance	*OsCBL10*	*calcineurin B-like protein10*	*Os01g0711500*	*LOC_Os01g51420*	Calcineurin B-like protein, calcium sensor, flooding response during seed germination	99.62% (265/266), 0% (0/266)	Ye et al. (2018)
Salt tolerance	*SIT1*	*SALT INTOLERANCE1*	*Os02g0640500*	*LOC_Os02g42780*	Lectin receptor-like kinase, mediation of salt sensitivity, regulation of ethylene homeostasis	98.37% (663/674), 0.15% (1/674)	Li et al. (2014)
Drought and salt tolerance	*DST*	*DROUGHT AND SALT TOLERANCE*	*Os03g0786400*	*LOC_Os03g57240*	C2H2 zinc finger transcription factor	93.57% (291/311), 4.50% (14/311)	Santosh Kumar et al. (2020)
Drought and salt tolerance	*DCA1*	*DST CO-ACTIVATOR 1*	*Os10g0456800*	*LOC_Os10g31850*	CHY zinc finger protein, transcriptional co-activator of DST	98.87% (263/266), 0% (0/266)	Cui et al. (2015)

Notes: ^1)^CGSNL = Committee on Gene Symbolization, Nomenclature and Linkage; ^2)^RAP = Rice Annotation Project. ^3)^MSU = Michigan State University. For the sequence of *O. longistaminata* see Reuscher et al. (2018)

In addition to the highly undesirable genes needing knock-out in order to produce agronomically acceptable phenotypes, a number of genes should also be edited in order to produce phenotypes which are targeted specific environments. These are genes related to heading date preventing *O. longistaminata* from flowering too early in its life cycle in short day environments and too late in higher latitudes (Table 2). There are also genes related to rooting depths, which may prove useful to construct deep-rooting phenotypes suitable for environments with a risk of drought during the growing cycle (Table 2). Finally, consumer preferences also differ widely and therefore genes involved in seed quality are also important target genes (Table 2).

**Table 2:** Key candidate genes to be knocked out in *Oryza longistaminata* in order to construct phenotypes suited for specific environments and consumer preferences

Trait	Gene name	CGSNL^1^ gene name or gene name synonym(s)	RAP^2^ ID	MSU^3^ ID	Description	Similarity and gaps to *O. sativa* [or haplotype]	References
Seed quality (fragrance)	*BADH2*	*betaine-aldehyde dehydrogenase 2*	*Os08g0424500*	*LOC_Os08g32870*	Betaine aldehyde dehydrogenase For fragrant rice: knockout	99.80% (500/501), 0% (0/501)	Bradbury et al. (2005), Chen et al. (2008); Kovach et al. (2009); Usman et al. (2020)
Eating and cooking quality	*Wx*	*WAXY*	*Os06g0133000*	*LOC_Os06g04200*	Granule-bound starch synthase, synthesis of amylose in endosperm. GT to TT mutation at the 5' splice site of 1st intron leds low amylose. For high amylopectin (sticky rice): knockout	[GT type]	Konishi et al. (2008), Li et al. (2022), Tian et al. (2009)
Eating and cooking quality	*SSII-3 (ALK)*	*SOLUBLE STARCH SYNTHASE 2–3* (*ALKALI DEGENERATION*)	*Os06g0229800*	*LOC_Os06g12450*	Soluble starch synthase II-3, endosperm starch synthesis GC/TT polymorphism at exon8 correlates rice varieties with high or intermediate gelatinization temperature (GT) (possessing the GC allele) and low GT (possessing the TT allele), respectively	[GC type]	Bao et al. (2006), Tian et al. (2009), Waters et al. (2006)
Grain shape	*GS9*	*GRAIN SHAPE GENE ON CHROMOSOME 9*	*Os09g0448500*	*LOC_Os09g27590*	Transcriptional activator For slender grains: knockout For round grains: Upregulation of expression	95.97% (333/347), 2.59% (9/347)	Lin et al. (2022), Zhao et al. (2018)
Grain shape	*GW7*	*GRAIN-WIDTH 7*	*Os07g0603300*	*LOC_Os07g41200*	TON1 RECRUIT MOTIF (TRM)-containing protein For grain width: knockout of GW7	94.04% (268/285), 2.81% (8/285)	Wang et al. (2015)
Grain shape	*GW8*	*GRAIN-WIDTH 8*	*Os08g0531600*	*LOC_Os08g41940*	SBP (SQUAMOSA promoter Binding Protein) DNA binding protein 16 For grain length: knockout of GW8	98.94% (931/941), 0.21% (2/941)	Wang et al. (2012)
Root system architecture	*DRO1*	*DEEPER ROOTING 1*	*Os09g0439800*	*LOC_Os09g26840*	IGT gene family, unknown function	99.60% (250/251), 0% (0/251)	Kitomi et al. (2020), Uga et al. (2013)
Root system architecture	*qSOR1 (DRL1)*	*QTL for SOIL SURFACE ROOTING 1* (*DRO1-LIKE 1*)	*Os07g0614400*	*LOC_Os07g42290*	Homolog of DRO1 For drought tolerance: upregulation of DRO1 expression and possession of WT qSOR1 For salinity tolerance: knockout of DRO1 and qSOR1	91.18% (279/306), 5.56% (17/306)	Kitomi et al. (2020), Uga et al. (2013)
Flowering time	*Hd2 (DTH7)*	*HEADING DATE2* (*Days to heading 7*)	*Os07g0695100*	*LOC_Os07g49460*	Pseudo response regulator, similar to two-component response regulator-like PRR37	99.19% (736/742), 0% (0/742)	Zhou et al. (2021)
Flowering time	*Hd5 (DTH8/Ghd8)*	*HEADING DAT5* (*Days to heading8/grain number plant height and heading date 8*)	*Os08g0174500*	*LOC_Os08g07740*	Putative HAP3 subunit of the CCAAT box-binding transcription factor, NF-YB11 (NUCLEAR FACTOR-Y subunit B11))	95.17% (276/290), 2.07% (6/290)	Zhou et al. (2021)
Flowering time	*Ehd1 (EF1)*	*EARLY HEADING DATE 1* (*EARLINESS 1*)	*Os10g0463400*	*LOC_Os10g32600*	B-type response regulator	98.24% (335/341), 0% (0/341)	Zhou et al. (2021)
Flowering time	*RFT1*	*RICE FLOWERING-LOCUS T 1*	*Os06g0157500*	*LOC_Os06g06300*	Florigen	98.88% (176/178), 0% (0/178)	Zhou et al. (2021)
Flowering time	*Hd3a (FT)*	*HEADING DATE 3A* (*Flowering locus T*)	*Os06g0157700*	*LOC_Os06g06320*	Florigen	100% (178/178), 0% (0/178)	Zhou et al. (2021)
Flowering time	*Hd6*	*HEADING DATE 6*	*Os03g0762000*	*LOC_Os03g55389*	Similar to protein kinase casein kinase II alpha subunit *O. longistaminata* possesses “Kasalath” type (functional)	(vs. “Kasalath”) 100% (333/333), 0% (0/333)	Takahashi et al. (2001)
Flowering time	*FRRP1*	*Flowering-Related RING Protein 1*	*Os10g0565600*	*LOC_Os10g41590*	E3 ligases of H2Bub1, C3HC4-type RING finger protein	99.41% (839/844), 0% (0/844)	Du et al. (2016)

^1^CGSNL = Committee on gene symbolization, nomenclature and linkage; ^2^RAP = rice annotation project. ^3^MSU = Michigan state university. For the sequence of *O. longistaminata* see Reuscher et al. (2018)

In the past, it was necessary to rely on gene transfer by transformation methods to achieve arbitrarily high expression of agriculturally useful genes. Recently, it has been reported that it is possible to introduce mutations in the cis region of a promoter to affect the timing and expression level of its downstream genes by introducing mutations in the cis region of the promoter through a multiplex system that can introduce mutations in multiple locations, based on SDN1 technology (Hendelman et al. 2021; Rodríguez-Leal et al. 2017). We have therefore also listed genes that are expected to improve the trait by high expression in *O. longistaminata* (Table 3).

**Table 3:** Key candidate genes in *Oryza longistaminata* to be targeted for introducing mutations in the cis region of the promoter

Trait	Gene name	CGSNL^1^ gene name or gene name synonym(s)	RAP^2^ ID	MSU^3^ ID	Description	Similarity and gaps to *O. sativa* [or haplotype]	References
Plant architecture	*MOC1*	*MONOCULM 1*	*Os06g0610300*	*LOC_Os06g40780*	GRAS protein 33	97.75% (434/444), 1.13% (5/444)	Li et al. (2003)
Seed quality	*GluA2*	*GLUTELIN SUBFAMILY A2 FROM WILD RICE SPECIES*	*Os10g0400200*	*LOC_Os10g26060*	Seed storage protein GLUTELIN	99.60% (498/500), 0% (0/500)	Yang et al. (2019)
Seed quality	*GIF1* (*CIN2*)	*GRAIN INCOMPLETE FILLING 1* (*cell-wall invertase 2*)	*Os04g0413500*	*LOC_Os04g33740*	Cell-wall invertase	98.17% (590/601), 0.50% (3/601)	Wang et al. (2008)
Grain shape	*PGL1* (*ILI6*)	*POSITIVE REGULATOR OF GRAIN LENGTH 1* (*INCREASED LEAF INCLINATION 6*)	*Os03g0171300*	*LOC_Os03g07510*	Atypical non-DNA-binding bHLH protein	100% (92/92), 0% (0/92)	Heang and Sassa (2012a, b)
Grain shape	*GS2*	*GRAIN SIZE 2*	*Os02g0701300*	*LOC_Os02g47280*	Growth-Regulating Factor 4 (GRF4)	98.41% (309/314), 0% (0/314)	Li et al. (2018)
Grain shape	*BG1*	*BIG GRAIN1*	*Os03g0175800*	*LOC_Os03g07920*	Positive regulator of auxin response and transport	98.08% (307/313), 1.28% (4/313)	Liu et al. (2015)
Grain shape	*LG1*	*LARGE GRAIN 1*	*Os02g0244300*	*LOC_Os02g14730*	Ubiquitin-specific protease 15, deubiquitination enzyme	99.69% (972/975), 0% (0/975)	Shi et al. (2019)
Grain shape	*GLW7*	*GRAIN LENGTH AND WEIGHT ON CHROMOSOME 7*	*Os07g0505200*	*LOC_Os07g32170*	SBP (SQUAMOSA promoter Binding Protein) DNA binding protein 13	95.50% (212/222), 2.70% (6/222)	Si et al. (2016)
Panicle architecture	*NOG1*	*NUMBER OF GRAINS 1*	*Os01g0752200*	*LOC_Os01g54860*	Enoyl-CoA hydratase/isomerase	99.74% (382/383), 0% (0/383)	Huo et al. (2017)
Panicle architecture	*DEP1*	*DENSE AND ERECT PANICLE 1*	*Os09g0441900*	*LOC_Os09g26999*	Unknown phosphatidylethanolamine-binding protein (PEBP) like domain protein	96.56% (421/436), 2.29% (10/436)	Huang et al. (2009), Sun et al. (2018)
Panicle architecture	*GGC2*	*-*	*Os08g0456600*	*-*	G Protein gamma subunit	97.33% (328/337), 0.59% (2/337)	Sun et al. (2018)
Nutrient uptake and photosynthesis	*AHA1* (*OSA1*)	*H* + *-ATPASE 1*	*Os03g0689300*	*LOC_Os03g48310*	Plasma membrane H^+^-ATPase 1, regulation of ammonium (NH_4_^+^) uptake	99.58% (952/956), 0% (0/956)	Zhang et al. (2021)

^1^CGSNL = committee on gene symbolization, nomenclature and linkage; ^2^RAP = rice annotation project. ^3^MSU = Michigan State University. For the sequence of *O. longistaminata* see Reuscher et al. (2018)

**Fig. 4:**
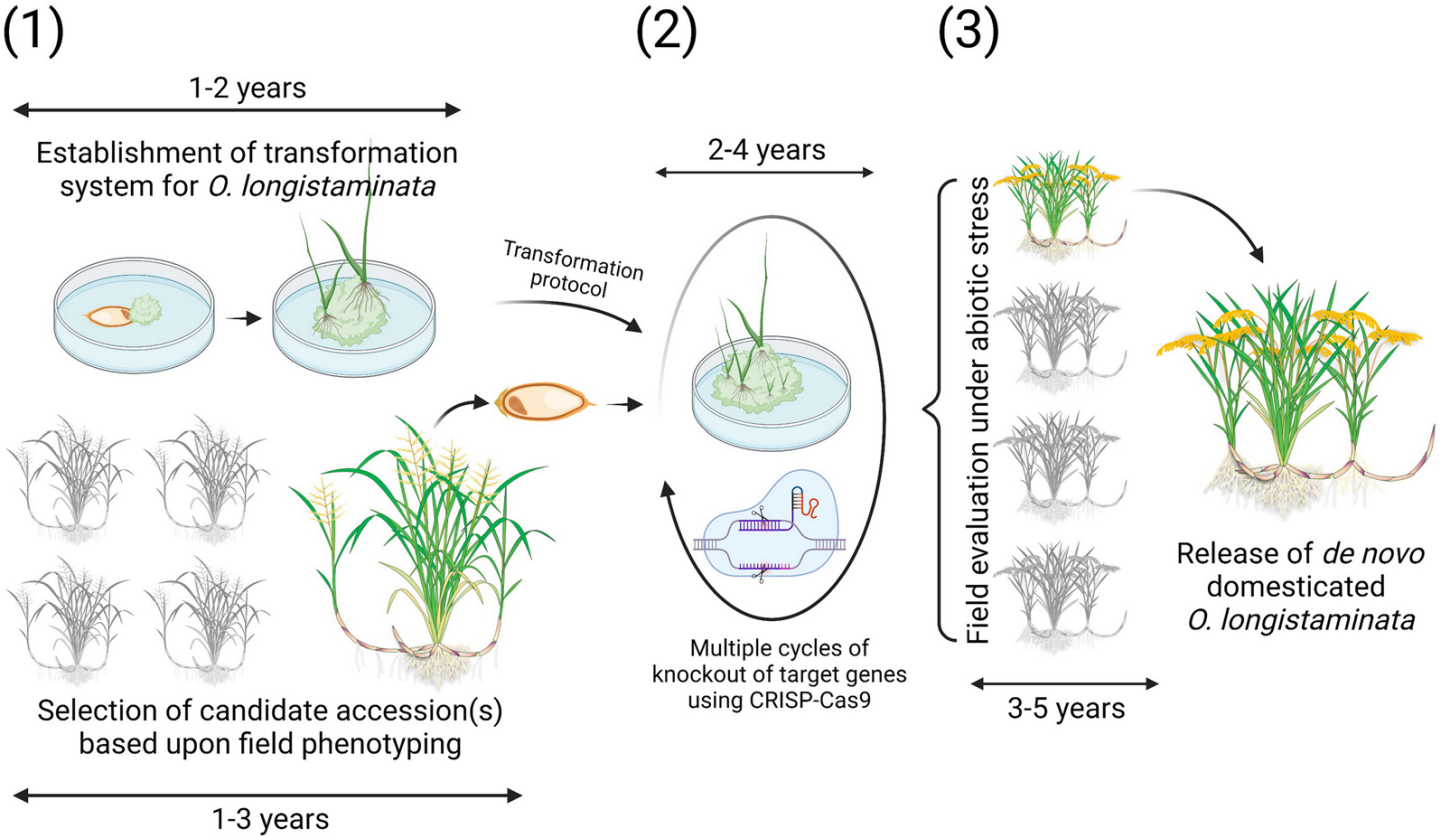
Timeline for de novo domestication of *Oryza longistaminata*. (1) establishment of a transformation system and selection of genotype(s) based on field performance under the relevant abiotic stress conditions, (2) multiple cycles of knockout of target genes, and (3) a final cycle of field evaluation in the relevant target environments. Created with BioRender.com

*O. longistaminata* has already been sequenced (Reuscher et al. 2018), and this is the first prerequisite for a successful de novo domestication (Abdullah et al. 2022). However, there are still several steps requiring evaluation before the actual work can begin. First, a transformation system needs to be established (Fig. 4), and the capability to induce callus and generate new plantlets is often the bottleneck to establish a transformation system (Abdullah et al. 2022). Conveniently, most of the fundamental steps have already been taken more than 30 years ago by a study reporting a protocol for plant regeneration from leaf and seed-derived calli and suspension cultures (Boissot et al. 1990). Recently, a more advanced system has been developed based on immature embryo rescuing in 11 species of wild rice, which also involved successful generation of callus of *O. longistaminata* (Shimizu-Sato et al. 2020). In parallel with establishing a transformation system, the target genotype can be identified. Fortunately, there are known accessions of *O. longistaminata*, which only show a minor degree of self-incompatibility (NBRP-Rice 2023), and these genotypes should be evaluated under the targeted abiotic stress (Fig. 4). Once these two initial steps are completed, the work involving knockout of undesirable target genes would require multiple cycles of gene editing using CRISP-Cas9 (Fig. 4). Finally, the new genotype(s) needs to undergo a thorough field evaluation in the environments for which the de novo domesticated *O. longistaminata* is targetted for (Fig. 4). In total, the process could take anything from 6 years, if substantial resources are allocated to the project, and up to 12 years with only minor resources available and/or with unforeseen bumps on the road (Fig. 4).

## Conclusion and Outlook

The traits needed for crops in future sustainable agriculture are already present in the natural vegetation, i.e., there is no need to reinvent the wheel. The current focus in rice breeding is on yield improvement, disease resistance and tolerance to abiotic stress, and valuable genes coding for these traits are present in the wild relatives of rice, including in *O. longistaminata*. Fortunately, new breeding techniques have made it feasible to accelerate domestication, which would otherwise take unacceptably long time utilizing traditional breeding, and there is therefore hope for green alternatives to our future food supply (Luo et al. 2022). *O. alta*, a wild rice species within the CCDD genome, is a showcase example of de novo domestication where genes responsible for contrasting traits such as seed shattering, plant height, and long heading date have been modified using genomic editing (Yu et al. 2021).

In Africa alone, 33% of the rice producing areas are prone to droughts with only 2% being affected by salinity (van Oort 2018), but the latter is expected to grow substantially in the future as a result of climate change (Shin et al. 2022). Therefore, climate-resilient alternatives to current rice cultivars are needed, and perennial cultivars requiring less input would be particularly attractive to small-hold farmers in the Global South. *O. longistaminata* holds a large potential for tolerance to abiotic stress including heat tolerance, drought tolerance and salinity tolerance, but it is also thought to be tolerant to lodging, and we therefore propose it being a suitable candidate species for de novo domestication. Wild rice does, however, possess several undesirable traits, including prostrate growth (Tan et al. 2008), high plant height (Zhang et al. 2020), long awn (Hua et al. 2015), seed shattering (Lin et al. 2007), and long heading date (Jing et al. 2018). Fortunately, a quick and efficient method to produce new rice germplasm resources is the directional modification of related genes in wild rice utilizing genome editing technologies (Gao 2021). For example, *HTD1* for plant architecture (Zou et al. 2006), *Gn1a* for yield (Ashikari et al. 2005), *SH1* and *SH4* for grain shattering (Konishi et al. 2006; Li et al. 2006), and *GS3* and *GW2* for seed size (Fan et al. 2009; Song et al. 2007) are all genes that have been shown to be of high agricultural value in rice. These genes in *O. longistaminata* can be mutated through genome editing to enable de novo domestication, and therefore *O. longistaminata* could be a good choice for the improvement of cultivated rice.

Currently, domestication is a continuous evolutionary process conducted by humans in four stages: *i*) the beginning of domestication, *ii*) the fixation of desirable alleles, *iii*) the generation of cultivated populations, and *iv*) selective breeding (Meyer and Purugganan 2013). However using de novo domestication, dot point *ii* “the fixation of desirable alleles” would be replaced by silencing of undesirable genes such as *sh4* and *prog1*, if present in the wild species.

## Data Availability

The data supporting the findings of this study are available from the corresponding authors, NK or OP, upon request.
